# Cardiac substrate metabolism in type 2 diabetes

**DOI:** 10.1042/BCJ20240189

**Published:** 2025-05-13

**Authors:** Jordan S. F. Chan, Tanin Shafaati, John R. Ussher

**Affiliations:** 1 Faculty of Pharmacy and Pharmaceutical Sciences, University of Alberta, Edmonton, AB, Canada; 2 Alberta Diabetes Institute, University of Alberta, Edmonton, AB, Canada; 3 Cardiovascular Research Institute, University of Alberta, Edmonton, AB, Canada

**Keywords:** cardiac substrate metabolism, diabetes, diabetic cardiomyopathy, diastolic dysfunction

## Abstract

As the most metabolically demanding organ on a per gram basis, substrate metabolism in the heart is intricately linked to cardiac function. Virtually all major cardiovascular pathologies are associated with perturbations in cardiac substrate metabolism, and increasing evidence supports that these perturbations in substrate metabolism can directly contribute to cardiac dysfunction. Furthermore, type 2 diabetes (T2D) is a major risk factor for increased cardiovascular disease burden, while also being characterized by a very distinct metabolic profile in the heart. This includes increases in cardiac fatty oxidation rates and a robust reduction in cardiac glucose oxidation rates. Herein, we will describe the primary mechanisms responsible for the increase in cardiac fatty acid oxidation and decrease in cardiac glucose oxidation during T2D, while also detailing perturbations in cardiac ketone and amino acid metabolism. In addition, we will interrogate preclinical studies that have addressed whether correcting perturbations in cardiac substrate metabolism may have clinical utility against ischemic heart disease, diabetic cardiomyopathy, or heart failure associated with T2D. Lastly, we will consider the translational potential of such an approach to manage cardiovascular disease in people living with T2D.

## Introduction

The heart is the most metabolically demanding organ in the body on a per gram basis, as it needs to continually produce adenosine triphosphate (ATP) to sustain pump function to ensure the body’s organs are readily supplied with oxygen (O_2_) and nutrients [1]. To meet its high energy demands, the heart acts as an omnivore and virtually utilizes any substrate that it has access to, which includes fatty acids, carbohydrates (i.e., glucose), ketone bodies (herein referred to as ketones), and amino acids [2,3]. Due to the heart’s reliance on substrate metabolism to support its function, it is now well accepted that perturbations in cardiac substrate metabolism can directly lead to cardiac pathologies.

One of the most relevant areas where this impact is felt lies within the global obesity epidemic. Increased adiposity and body weight gain are risk factors for insulin resistance and type 2 diabetes (T2D). T2D is characterized by insulin resistance, which often results in hyperinsulinemia as islet β-cells produce and secrete excess insulin as an adaptive response to limit hyperglycemia, though it has also been demonstrated that hyperinsulinemia can occur first and precipitate systemic insulin resistance [4]. As adipose mass expands and adipocytes enlarge, this results in elevated secretion of pro-inflammatory cytokines and a chronic low-grade inflammation, which further contributes to the hyperglycemia and insulin resistance that characterizes T2D [5]. Importantly, all of these independently increase the risk for underlying cardiovascular disease. Furthermore, the dyslipidemia, insulin resistance, and hormonal changes that manifest during T2D have profound actions on cardiac substrate metabolism [1,6].

In this review, we aim to describe the regulation of substrate metabolism in the healthy heart, while defining the major changes that take place during obesity and/or T2D. Our focus will primarily be in the realm of cardiac fatty acid and glucose metabolism, as these are the two major fuel sources for the heart that have been most extensively studied in the context of obesity/T2D. Nonetheless, the heart is also an avid consumer of ketones and remains capable of oxidizing amino acids; thus, we will also mention metabolic perturbations related to these fuel sources where appropriate. As perturbations in cardiac substrate metabolism can contribute to T2D-related cardiovascular disease, we will also consider the potential for metabolic approaches to treat ischemic heart disease, heart failure, and diabetic cardiomyopathy (DbCM) associated with T2D. We will primarily focus on preclinical studies, as many of the metabolic therapies within this realm are still in experimental stages, though we will also discuss the translational potential of these approaches in clinical studies. While many of the metabolic perturbations associated with T2D are also present in type 1 diabetes and may contribute to type 1 diabetes-associated cardiomyopathy, we will not cover this in any detail and encourage the reader to explore other reviews that have interrogated this topic [7–9].

## Substrate metabolism in the healthy heart

As previously stated, the heart is the most metabolically demanding organ in the body on a per gram basis. Thus, the regulation of cardiac substrate metabolism is complex and multifaceted [1]. A key determinant of rates of substrate metabolism is dependent on the circulating concentrations the heart is exposed to, which is further influenced by hormonal regulation. Following nutrient ingestion, carbohydrates from the meal stimulate insulin secretion, which increases cardiac glucose uptake and subsequent oxidation, while consequently decreasing fatty acid oxidation [10]. Conversely, increased circulating levels of glucagon during fasting enhance cardiac fatty acid oxidation while decreasing glucose oxidation. This reciprocal relationship between glucose and fatty acid oxidation is known as the ‘Glucose-Fatty Acid’ cycle and was described by Philip Randle and colleagues in 1963 [11], though first reported by Joseph Shipp and colleagues a few years prior [12]. Despite this competition between carbohydrates and fats for their respective consumption, the healthy heart still preferentially uses fatty acids such as palmitate and oleate to meet its energy demands, accounting for approximately 40–60% of total cardiac ATP production [13,14].

### Fatty acid metabolism

**Figure 1:**
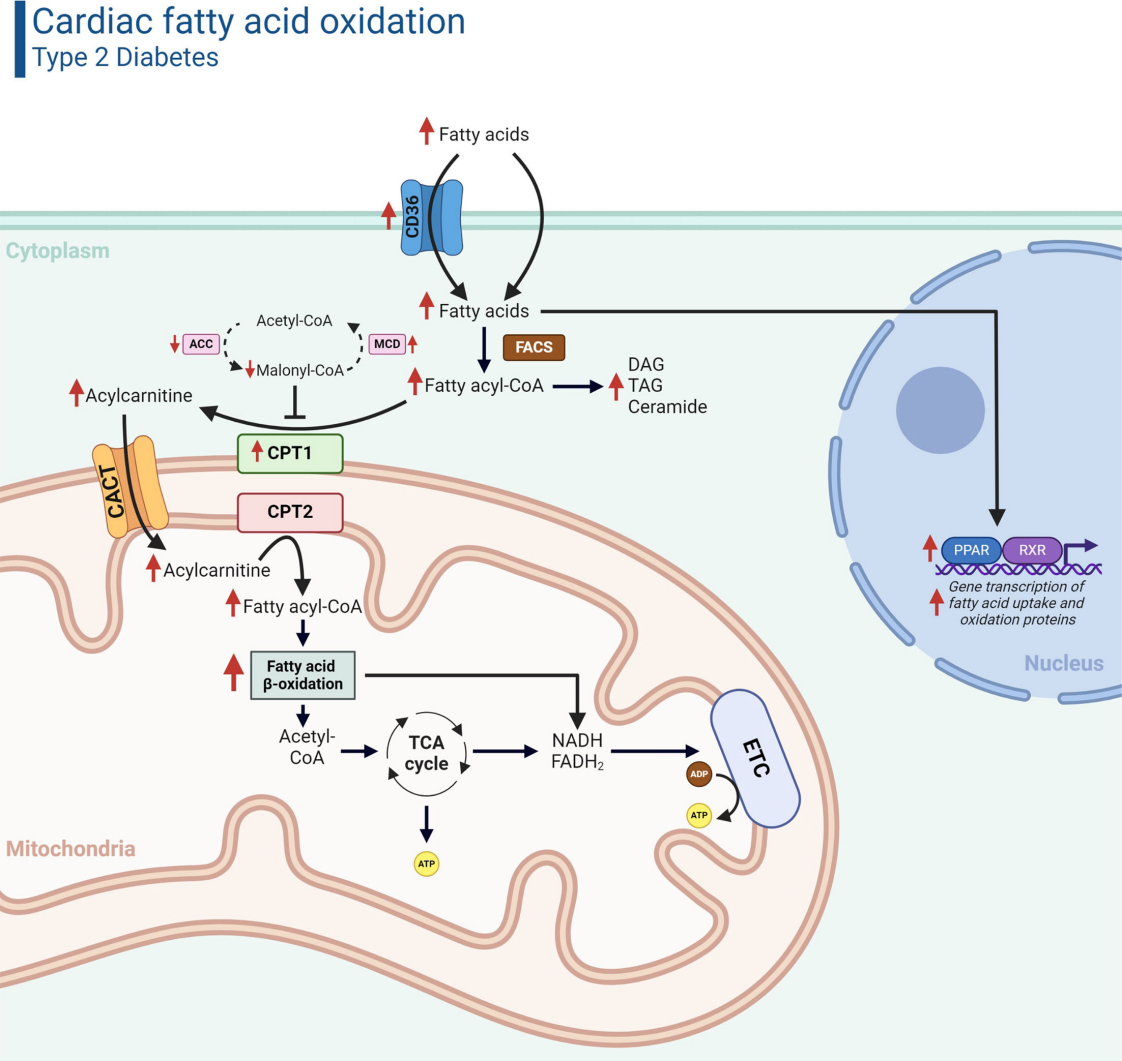
Cardiac fatty acid metabolism in T2D. The heart increasingly relies on fatty acid oxidation for energy production in the setting of T2D. Elevated intracellular fatty acid levels due to persistent hyperlipidemia increases the transcriptional activity of PPAR/RXR, which in turn up-regulates the gene transcription of several proteins involved in fatty acid uptake and fatty acid oxidation (as indicated by the red arrows). ACC, acetyl CoA carboxylase; ADP, adenosine diphosphate; ATP, adenosine triphosphate; CACT, carnitine-acylcarnitine translocase; CD36, cluster of differentiation 36; CPT, carnitine palmitoyltransferase; DAG, diacylglycerol; ETC, electron transport chain; FACS, fatty acyl CoA synthetase; FADH_2_, flavin adenine dinucleotide; MCD, malonyl CoA decarboxylase; NADH, nicotinamide adenine dinucleotide; PPAR, peroxisome proliferator-activated receptor; RXR, retinoid X receptor; T2D, type 2 diabetes; TAG, triacylglycerol; TCA, tricarboxylic acid. Created in Biorender.com.

Nonesterified fatty acids bound to albumin, or lipoprotein lipase (LPL) liberated fatty acids from triacylglycerol (TAG) within chylomicrons or very low-density lipoproteins, reach cardiomyocytes through the coronary vasculature, and are primarily transported across the sarcolemmal membrane via protein-mediated transport or passive diffusion (Figure 1) [15]. Regarding the former, one of the major protein transporters that has been shown to mediate ~50% of cardiac fatty acid uptake is cluster of differentiation 36/fatty acid translocase (CD36/FAT). Fatty acids taken up into cardiomyocytes are then esterified to coenzyme A (CoA) via fatty acyl CoA synthetase (FACS) and subsequently converted into their respective fatty acylcarnitine by carnitine palmitoyltransferase 1 (CPT1) (Figure 1). This is a necessary step that allows fatty acids to traverse the inner mitochondrial membrane, which long-chain fatty acyl CoAs are impermeable against [16]. CPT2 converts the fatty acylcarnitine back into fatty acyl CoA in the mitochondrial matrix, after which it undergoes repeated cycles of fatty acid β-oxidation to produce acetyl CoA. The resulting acetyl CoA enters the tricarboxylic acid (TCA) cycle, producing reducing equivalents (i.e., nicotinamide adenine dinucleotide (NADH) and flavin adenine dinucleotide (FADH_2_)) that can be used to support ATP production via oxidative phosphorylation [17].

Metabolic flux through fatty acid β-oxidation is tightly regulated in the heart to maintain its ability to switch between different energy substrates, which has been extensively reviewed [15]. This includes regulation of fatty acid supply, fatty acid uptake, CPT1 activity via malonyl-CoA-mediated inhibition [18], as well as transcriptional and post-translational modification of key enzymes involved in fatty acid oxidation. Malonyl CoA levels are also tightly controlled at a molecular level, whereby malonyl CoA is synthesized by acetyl CoA carboxylase (ACC) and degraded via malonyl CoA decarboxylase (MCD) (Figure 1). Although fatty acids produce the greatest ATP yield per mol of substrate relative to other fuel sources, they have the highest O_2_ consumption, and as a result, fatty acids are considered to be the least ‘efficient’ fuel source in terms of O_2_ requirements (ATP produced/O_2_ consumed) [19].

### Glucose metabolism

**Figure 2:**
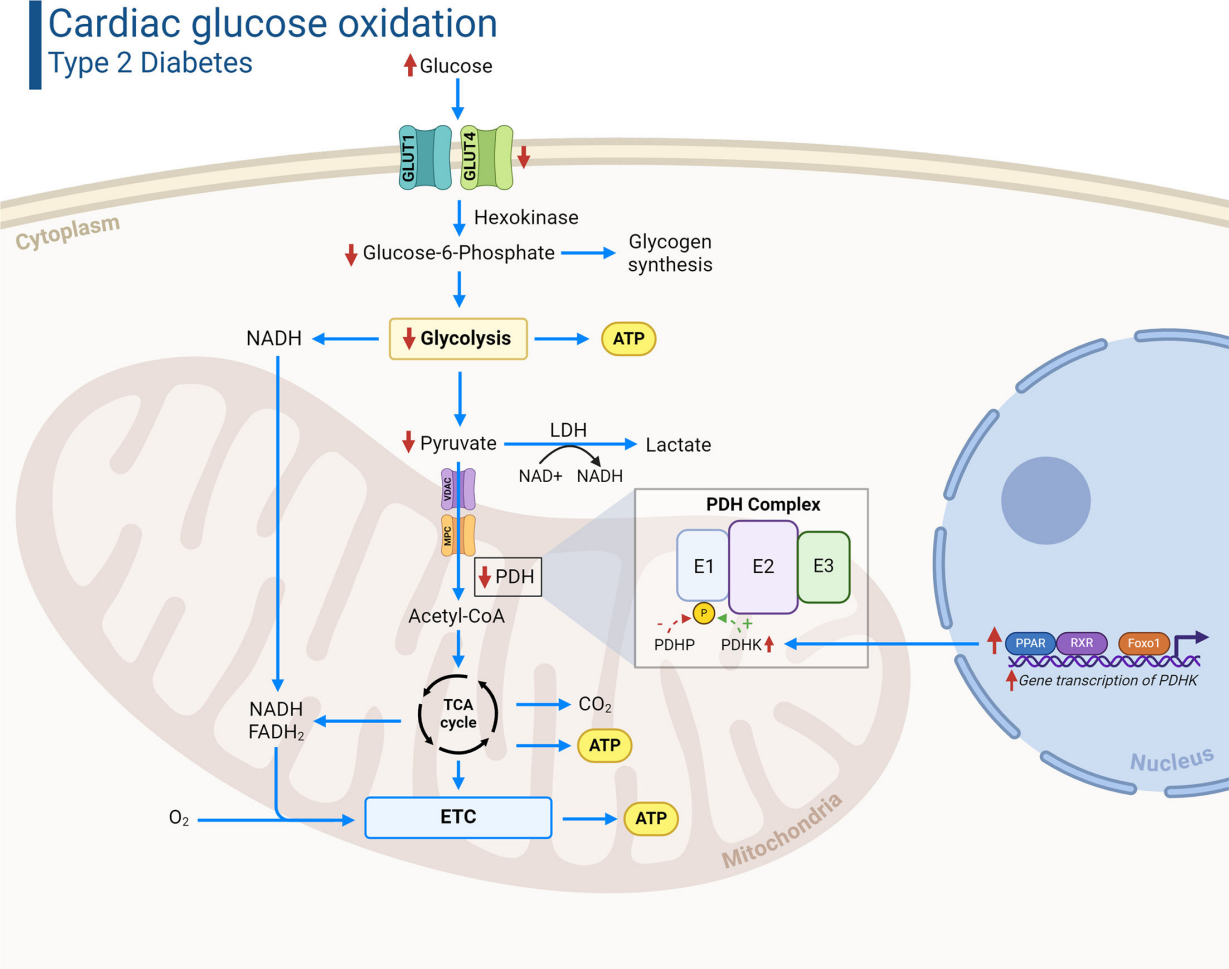
Cardiac glucose metabolism in T2D*.* Despite the increased circulating glucose levels in T2D, cardiac glucose uptake is generally diminished due to decreased GLUT4 expression and insulin resistance which reduces insulin-mediated GLUT4 translocation to the sarcolemmal membrane. Furthermore, the activity of the rate-limiting enzyme of glucose oxidation, PDH, is reduced secondary to increased inhibitory phosphorylation mediated by PDHK isoforms (primarily PDHK4). Elevated cardiac PPAR and FoxO1 activity, which is often observed in T2D, can increase the transcription of PDHK and consequently suppress PDH activity. Red arrows indicate the changes in metabolic flux and activity that occur in T2D. ATP, adenosine triphosphate; ETC, electron transport chain; FADH_2_, flavin adenine dinucleotide; FoxO1, forkhead box O1; GLUT, glucose transporter; LDH, lactate dehydrogenase; MPC, mitochondrial pyruvate carrier; NAD^+^/NADH, nicotinamide adenine dinucleotide; PDH, pyruvate dehydrogenase; PDHK, pyruvate dehydrogenase kinase; PDHP, pyruvate dehydrogenase phosphatase; PPAR, peroxisome proliferator-activated receptor; RXR, retinoid X receptor; T2D, type 2 diabetes; TCA, tricarboxylic acid; VDAC, voltage-dependent anion-selective channel. Created in Biorender.com.

Glucose represents the second main fuel source that the heart consumes and accounts for 20–40% of ATP production, though it becomes a more prominent fuel source following ingestion of a carbohydrate-containing meal [14]. Glucose enters the cardiomyocyte through GLUT1 (basal) and GLUT4 (insulin sensitive) transporters, upon which it is immediately phosphorylated by hexokinase to glucose-6-phosphate (G6P), due to hexokinase’s high affinity for glucose (Figure 2). G6P is a necessary intermediate for several metabolic fates of glucose, including glycolysis, glycogen synthesis, and the pentose phosphate pathway, the latter of which is negligible in the heart [12]. Glycolysis ultimately results in the production of pyruvate, which, in an aerobic environment, is transported into the mitochondrial matrix through a monocarboxylic acid transporter (MCT) known as the mitochondrial pyruvate carrier (MPC). The majority of pyruvate is then converted into acetyl CoA by pyruvate dehydrogenase (PDH), the rate-limiting enzyme of glucose oxidation, following which it enters the TCA cycle to generate NADH and FADH_2_ for oxidative phosphorylation and ATP production (Figure 2) [20]. To a lesser extent, pyruvate can also enter the TCA cycle via alternative fates to support anaplerosis, including pyruvate carboxylase-mediated conversion into oxaloacetate, or malic enzyme-mediated conversion into malate [21].

Activity of the PDH complex is tightly regulated by several factors, including reversible phosphorylation of enzyme 1 of the PDH complex (PDH itself) by PDH kinase (PDHK) and PDH phosphatase (PDHP). The former is responsible for phosphorylation/inactivation, while the latter dephosphorylates/activates the PDH complex [22]. In contrast with fatty acids, glucose is the more ‘efficient’ fuel source in terms of O_2_ requirements (ATP produced/O_2_ consumed) [19], which may have therapeutic utility in relation to T2D-related cardiovascular disease.

### Ketone and amino acid metabolism

**Figure 3:**
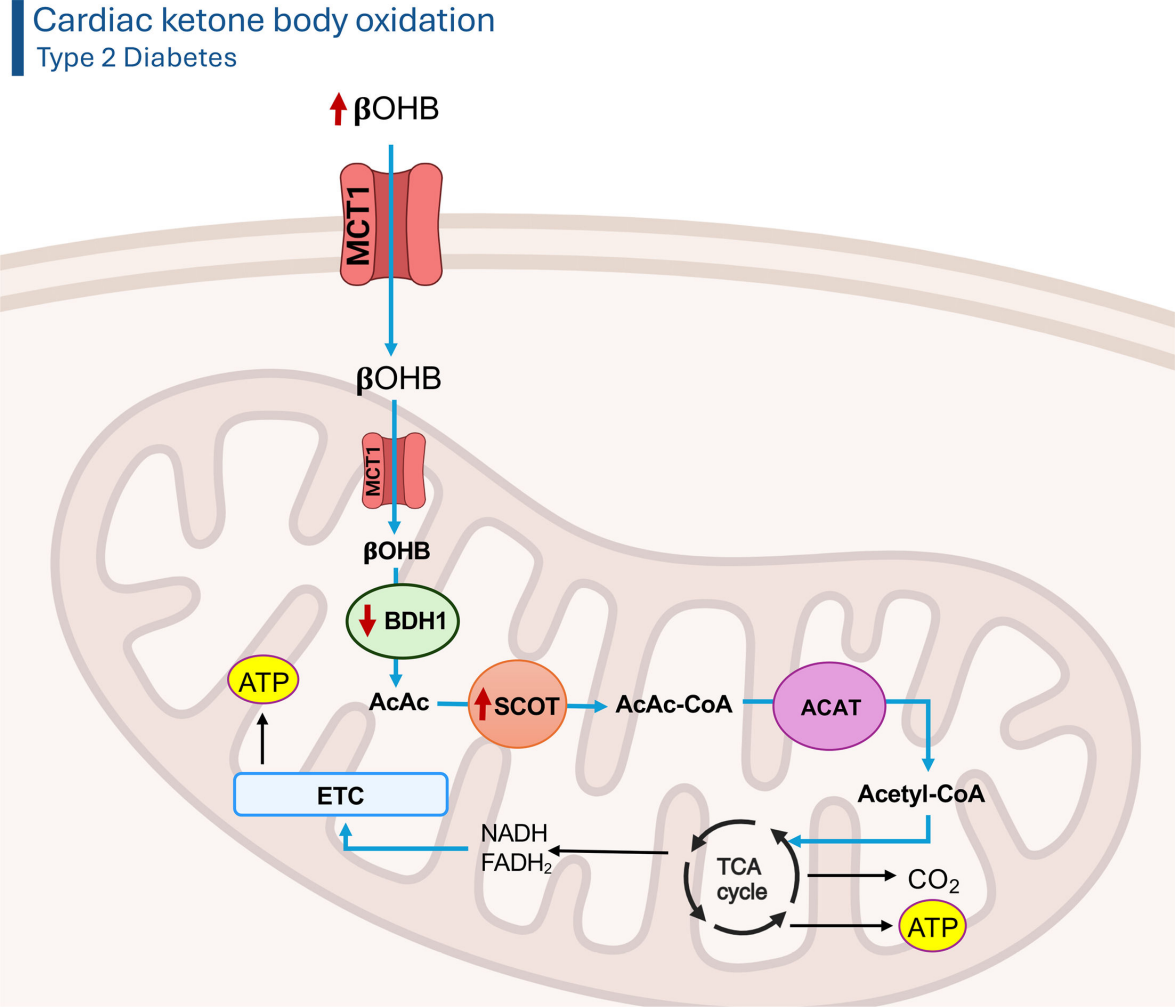
Cardiac ketone metabolism in T2D*.* During T2D, there is a down-regulation of BDH1 protein expression and an up-regulation of SCOT activity (as indicated by the red arrows), which may explain contrasting findings on whether ketone oxidation is decreased or increased in the diabetic heart depending on which ketone substrate is being provided (AcAc or βOHB). AcAc, acetoacetate; AcAc-CoA, acetoacetyl CoA; ACAT, acetoacetyl CoA thiolase; ATP, adenosine triphosphate; βOHB, β-hydroxybutyrate; BDH1, β-hydroxybutyrate dehydrogenase 1; ETC, electron transport chain; FADH_2_, flavin adenine dinucleotide; MCT1, monocarboxylic acid transporter 1; NADH, nicotinamide adenine dinucleotide; SCOT, succinyl CoA:3-ketoacid CoA transferase; T2D, type 2 diabetes; TCA, tricarboxylic acid. Created in Biorender.com.

The regulation of ketone and amino acid metabolism in the heart has been comparatively understudied versus that of fatty acids and carbohydrates, though we encourage the reader to read the following for extensive review of the molecular regulation of ketone metabolism in the heart [23,24]. Circulating ketones primarily comprise acetoacetate and β-hydroxybutyrate (βOHB), both of which are taken up into cardiomyocytes via either passive diffusion or transported through MCTs [24,25]. Following the conversion of βOHB to acetoacetate via βOHB dehydrogenase 1 (BDH1), succinyl CoA:3-ketoacid CoA transferase (SCOT), the rate-limiting enzyme of ketone oxidation, activates acetoacetate for subsequent oxidation via esterifying it to CoA and generating acetoacetyl CoA (Figure 3) [24,25]. Unlike the activation of a fatty acid to fatty acyl CoA or glucose to G6P, the activation of acetoacetate to acetoacetyl CoA does not require ATP, and acetoacetyl CoA is subsequently hydrolyzed via acetoacetyl CoA thiolase to acetyl CoA for entry into the TCA cycle. Ketones are an important fuel source for the heart, especially during fasting/starvation, with studies suggesting that they account anywhere from 10% to 20% of overall ATP production [23]. Studies in pigs have suggested that increased ketone delivery to the heart can lead to a corresponding decline in cardiac fatty acid oxidation, mirroring a Randle cycle-like substrate competition [26]. In contrast, increasing the concentration of the ketone, βOHB, in the Krebs Henseleit perfusate did not affect fatty acid or glucose oxidation rates in aerobically perfused isolated working mouse hearts [27,28]. Whether these differences can be explained by species-specific or methodological-specific differences remains unknown, and further research is necessary to determine how ketones affect cardiac substrate metabolism.

In contrast to fatty acids, carbohydrates, and ketones, most evidence suggests that amino acids provide negligible amounts toward overall oxidative ATP production. In particular, the branched-chain amino acids (leucine, isoleucine, and valine; BCAAs), which represent some of the most extensively studied amino acids with regard to intermediary metabolism, account for only 1% of overall ATP production in aerobically perfused isolated working mouse hearts and are unresponsive to the actions of insulin [29]. Although amino acids are not significant sources of ATP production for the heart, several amino acids serve as key anaplerotic substrates that ensure optimal flux through the Krebs cycle [30].

## Substrate metabolism in the heart in the setting of T2D

### Experimental models of T2D

Most of our understanding regarding how T2D affects cardiac substrate metabolism has been derived from animal studies, though many of the major metabolic findings in diabetic animals have since been recapitulated in human studies. Several genetic and dietary models are available to study insulin resistance, prediabetes, and/or T2D in animals [31]. The primary ones that have been utilized to interrogate cardiac substrate metabolism include *db*/*db* mice, which exhibit robust obesity, insulin resistance, and a T2D phenotype secondary to spontaneous mutation in the leptin receptor, as well as diet-induced obesity via consumption of a high-fat diet (HFD), which causes insulin resistance and prediabetes in mice. In addition, experimental T2D can be induced in rodents via a combination of HFD feeding (8 weeks or greater) with a low-dose injection of the β-cell toxin, streptozotocin (STZ), which eliminates vulnerable β-cells. Although each of these models offer several advantages and disadvantages with regard to modeling T2D and their capacity to yield cardiac dysfunction in animals, which has been extensively reviewed by others [32], studies using these three models for most part have reported comparable actions on cardiac substrate metabolism.

### Fatty acid metabolism

The heart loses its metabolic flexibility in obesity and/or T2D, driven in part by cardiac insulin resistance, resulting in an increased reliance on fatty acid oxidation for ATP production and a corresponding decrease in cardiac glucose oxidation [33]. These perturbations in cardiac energy metabolism are well documented in both patients with obesity/T2D and in preclinical studies. Positron emission tomography (PET) imaging studies have demonstrated that patients with T2D have increased cardiac fatty acid uptake and oxidation relative to nondiabetic control subjects [34,35]. Although this was associated with impaired cardiac efficiency [34], these impairments were not directly correlated with left ventricular (LV) diastolic function [35]. Conversely, magnetic resonance spectroscopy (MRS) imaging studies in patients with T2D revealed an association between increased myocardial TAG content and diastolic dysfunction as evidenced by reductions in the E (blood flow velocity during early diastole)/A (blood flow velocity during late diastole) ratio and E peak deceleration [36]. A plethora of preclinical studies have also observed elevated cardiac fatty acid oxidation rates in animal models of obesity/T2D using isolated working heart perfusions with radiolabeled palmitate or oleate, including *db*/*db* mice, HFD-fed mice, and HFD-fed plus low-dose STZ-treated mice [37–40].

It should be noted that other studies have reported that obesity/T2D can be associated with reductions in cardiac fatty acid oxidation. For example, oleate oxidation rates are decreased during isolated working heart perfusions from fasted male obese Zucker rats [41], while mitochondria from permeabilized LV fibers of individuals with T2D demonstrated decreased O_2_ consumption in the presence of palmitoylcarnitine [42]. However, these studies have important limitations that need to be considered. This includes the obese Zucker rat heart perfusions being done in the presence of low oleate concentrations (0.4 mM), where it has been demonstrated that significant TAG mobilization and endogenous fatty acid oxidation rates are increased [43]. As obese Zucker rat hearts have a marked elevation in TAG content, it is highly likely that endogenous fatty acid oxidation rates were elevated in those studies, thereby underestimating overall cardiac fatty acid oxidation rates. Moreover, a combination of PET imaging with [1-^11^C]palmitate and [^1^H] magnetic resonance reported that endogenous fatty acid oxidation rates are elevated in obese and insulin-resistant individuals [44]. Regarding the permeabilized LV fiber studies, as these originated from individuals with T2D and stable coronary artery disease, the latter of which would be associated with reduced overall oxidative metabolism due to myocardial ischemia, it could explain the observed reduction in palmitoylcarnitine supported O_2_ consumption. There are also profound differences in workload from isolated mitochondria versus the *ex vivo* working or intact heart that need to be considered when comparing various methods to assess cardiac substrate metabolism [1]. Hence, the vast majority of human and rodent studies using radioisotopes via PET imaging or working heart perfusions that have observed increased cardiac fatty acid oxidation rates in obesity/T2D are likely the more accurate findings.

Several molecular mechanisms have been identified that contribute to the increased cardiac fatty acid oxidation in the setting of obesity/T2D, with one of the most important being an increase in transcriptional activity of peroxisome proliferator-activated receptor-α (PPARα). Elevated cardiac PPARα activity induces the transcription of CD36/FAT, and there is also increased translocation of CD36/FAT from endosomes to the sarcolemmal membrane, thereby facilitating excess fatty acid uptake into the cardiomyocyte [45,46]. PPARα also increases the transcription of fatty acid-binding protein and FACS, promoting the activation of fatty acids for subsequent metabolism, while also increasing the transcription of MCD and CPT1, ensuring that activated fatty acids have unimpeded transport into the mitochondria. Lastly, PPARα increases the transcription of key enzymes of fatty acid β-oxidation such as medium-chain acyl CoA dehydrogenase and long-chain acyl CoA dehydrogenase [15]. PPARα may also stimulate TAG turnover by regulating transcription of acyltransferases (e.g., diacylglycerol acyltransferase) and lipases (e.g., adipose tissue TAG lipase (ATGL)) in the heart in obesity/T2D [47]. This could be metabolically wasteful as mobilized fatty acids from TAG have to be re-esterified to CoA via FACS prior to entering the mitochondria for β-oxidation.

Transgenic mice with a cardiac-specific overexpression of PPARα developed cardiac hypertrophy and ventricular dysfunction, which suggests that the above-described metabolic perturbations are maladaptive in obesity/T2D [48]. Similarly, it has also been demonstrated that increase reliance on fatty acids as an energy source increases reactive oxygen species production [49] and mitochondrial membrane uncoupling [50], while negatively affecting cardiac efficiency [39].

Despite the increased cardiac fatty acid oxidation observed in obesity/T2D, excess cardiac lipid accumulation and lipotoxicity have also been observed in the heart, likely indicating that cardiac fatty acid supply and uptake outpace the increase in fatty acid oxidation. This may also be driven by PPARα, as studies by Nakamura and colleagues have demonstrated that a specific PPARα transcriptional signature regulated by glycogen synthase kinase 3α (GSK3α) contributes to cardiac lipid accumulation and lipotoxicity in obesity/T2D [51]. In particular, they observed that dyslipidemia in mice fed a HFD up-regulates GSK3α activity, which increases PPARα phosphorylation at serine 280 (Ser280), thereby increasing gene transcription of CD36/FAT and fatty acid transport protein 1, but not PPARα-regulated genes involved in enhancing fatty acid oxidation. Increases in LPL activity also contribute to increased cardiac fatty acid uptake during obesity/T2D [52]. This involves several mechanisms, whereby insulin resistance leads to reductions in vascular endothelial growth factor B expression, as well as enhanced LPL delivery to the coronary lumen via increased endothelial cell heparanase activity, both of which contribute to increased cardiac fatty acid oxidation and lipotoxicity [53,54]. Accordingly, extensive research has been dedicated to the development of therapies that can attenuate cardiac fatty acid uptake and subsequent oxidation, or alleviate lipotoxicity as a strategy for improving cardiac function in obesity/T2D, which we will discuss in the ensuing sections.

### Glucose metabolism

As previously stated, enhanced cardiac fatty acid oxidation rates in obesity/T2D result in a corresponding suppression of glucose oxidation due to the Randle cycle phenomenon [11]. PET imaging studies using ^18^F-2-fluoro-2-deoxy-D-glucose (^18^FDG) revealed decreased cardiac glucose uptake in both male and female participants with T2D relative to lean volunteers [35,55,56]. These observations were further supported by hyperpolarized ^13^C MRS studies demonstrating decreased enzymatic flux through PDH in participants with T2D, as reflected by a reduction in [^13^C]bicarbonate production from [^13^C] pyruvate [57]. Isolated working heart perfusions conducted in rodent models of obesity/T2D have recapitulated the robust impairment in cardiac glucose oxidation observed in humans [40,58,59]. Complementing these *ex vivo* experiments, *in vivo* ^13^C MRS imaging studies in male Wistar rats with T2D demonstrated a threefold decrease in cardiac PDH flux relative to their lean, nondiabetic counterparts [60]. Conversely, studies in low-density lipoprotein receptor knockout (KO) mice fed an HFD observed increases in *in vivo* cardiac glucose uptake assessed via ^18^FDG PET imaging, though this is likely attributed to the significant hyperinsulinemia associated with this model [61]. Moreover, ^18^FDG PET imaging is only indicative of glucose uptake rates and not reflective of actual glucose oxidation.

Mechanisms responsible for the decrease in cardiac glucose oxidation may be secondary to insulin resistance, which reduces insulin-mediated translocation of GLUT4 and subsequent glucose uptake into the cardiomyocyte [62]. In addition, cardiac GLUT4 expression is also reduced in obesity/T2D, further contributing to decreased glucose utilization [63,64]. Reductions in cardiac glucose oxidation are primarily driven by a decrease in PDH activity, often as a result of increased inhibitory phosphorylation mediated via PDHK4. This can be partly explained via the Randle cycle effect, as increased fatty acid oxidation-derived acetyl CoA and NADH levels increase PDHK and decrease PDHP activity, respectively [65]. With regard to molecular control, increased cardiac PPARα activity is thought to be a key player here, stemming from its ability to increase the transcription of *Pdk4* (gene encoding for PDHK4) [48,66]. Elevated cardiac PPARα activity also suppresses PDH activity by limiting mitochondrial matrix Ca^2+^ uptake via the up-regulation of the mitochondrial calcium uniporter complex inhibitory subunit (MCUb) [67]. Studies have also illustrated that the transcription factor forkhead box O1 (FoxO1) is hyperactive in the hearts of obese mice [68], which can also increase *Pdk4* transcription [69]. It is now increasingly recognized that other post-translational modifications of PDH such as acetylation and glutathionylation are elevated in the setting of obesity [70], which can also contribute to the impairment of cardiac PDH activity [71,72]. Yet, no study to date has compared the relative importance of these various post-translational modifications toward the overall regulation of PDH activity, and whether there may be relevant interactions between them.

In support of impaired PDH activity and cardiac glucose oxidation being detrimental to cardiac function, mice with a cardiac-specific KO of PDH (PDH^CardiacKO^) present with a DbCM-like phenotype [73]. Compared with their control littermates, male PDH^CardiacKO^ mice exhibited a near complete abolishment in cardiac glucose oxidation rates as determined by isolated working heart perfusions, while these animals showed signs of diastolic dysfunction (decreased mitral E/A ratio during pulsed-wave Doppler echocardiography) and cardiac hypertrophy.

### Ketone and amino acid metabolism

Although the impact of obesity and/or T2D on cardiac ketone metabolism has been minimally studied, isolated working hearts from male mice with experimental T2D (via HFD feeding in combination with a low-dose injection of STZ) demonstrated a marked reduction in βOHB oxidation rates [74]. Similarly, βOHB oxidation rates are decreased in isolated working hearts from male *db*/*db* mice [75]. Conversely, ^13^C MRS studies with hyperpolarized [3-^13^C]acetoacetate reported increases in cardiac ketone oxidation in the Goto-Kakizaki rat model of T2D [76]. A possible explanation for these discrepant findings may relate to the use of either acetoacetate or βOHB as the ketone substrate. Cardiac protein expression of BDH1 (converts βOHB into acetoacetate; Figure 3) is decreased in mice with HFD/low-dose STZ-mediated T2D [74], whereas cardiac enzymatic activity of SCOT (converts acetoacetate into acetoacetyl CoA; Figure 3) is increased in both mice with HFD/low-dose STZ-mediated T2D and Goto-Kakizaki rats [74,76]. Hence, it is not surprising that studies that use acetoacetate as the ketone substrate would report increases in cardiac ketone oxidation in experimental models of T2D, since acetoacetate would bypass BDH1 and serve as immediate substrate for up-regulated SCOT activity. In order to confirm this, future studies that directly compare βOHB and acetoacetate radioisotopes within the same experimental model of T2D are necessary.

There has been minimal interrogation as to how obesity and/or T2D affect the metabolism of the various amino acids in the heart. Despite BCAAs accounting for ~1% of overall cardiac ATP production, it has been observed that BCAA oxidation rates are decreased in isolated working hearts from mice subjected to experimental obesity via HFD feeding [29].

## Targeting cardiac substrate metabolism to treat ischemic heart disease in T2D

### Fatty acid metabolism

Despite the general reduction in oxidative metabolism resulting from myocardial ischemia, studies in isolated working rat hearts have demonstrated that fatty acids predominate as the primary fuel for residual oxidative metabolism in the ischemic heart [77]. These metabolic alterations in fatty acid metabolism are postulated to be harmful to cardiac function of the ischemic heart, since fatty acids are the less O_2_ efficient fuel [78], while high rates of fatty acid oxidation can also lead to cardiac acidosis by uncoupling glycolysis from glucose oxidation [79]. In support of this, inhibiting cardiac fatty acid oxidation often results in cardioprotection against ischemia/reperfusion injury. For example, direct treatment of isolated working male rat hearts with trimetazidine, which deceases fatty acid oxidation via inhibiting the β-oxidation enzyme, 3-ketoacyl CoA thiolase (3-KAT), improves cardiac functional recovery (e.g., cardiac output × peak systolic pressure) during reperfusion following a 30-min global no-flow ischemia [80]. Similar observations have been reported following inhibition of either CPT1 [81] or MCD [82] to decrease mitochondrial fatty acid uptake and subsequent oxidation in isolated working rat hearts. These observations have been recapitulated in genetic studies, as isolated working hearts from male whole-body MCD KO mice also demonstrated improved cardiac functional recovery (e.g., heart rate × peak systolic pressure) during reperfusion following a 20-min global no-flow ischemia [83].

Despite these positive outcomes, it should be noted that these studies were conducted in nondiabetic animal models of ischemic heart disease. Nonetheless, several studies from Aasum and colleagues have observed similar findings in mouse models of obesity/T2D. This includes treatment of *db*/*db* and HFD fed mice with rosiglitazone or fenofibrate, respectively, both of which improved reperfusion recovery of cardiac function (e.g., cardiac output and LV developed pressure) following a 40-min low-flow ischemia [84,85]. Although rosiglitazone and fenofibrate are agonists of PPARs and, on the surface, should increase cardiac fatty acid oxidation, due to their systemic actions on adipose and/or liver fatty acid metabolism, these agents decreased cardiac fatty acid oxidation likely secondary to a reduction in circulating fatty acid levels. Moreover, decreasing fatty acid oxidation via limiting fatty acid uptake with the CD36 inhibitor, sulfo-*N*-succinimidyl oleate, also enhanced the recovery of cardiac function (e.g., rate pressure product) in isolated working hearts from male Wistar rats with T2D (via combination of HFD feeding plus low-dose STZ injection) subjected to hypoxia/reoxygenation [86]. Importantly, these benefits in *ex vivo* models of ischemia/reperfusion injury appear to translate to *in vivo* models of acute myocardial infarction. An 8-day treatment with rosiglitazone (3 mg/kg via oral gavage) in male Zucker diabetic fatty rats produced smaller infarct sizes following a 30-min temporary left anterior descending (LAD) coronary artery occlusion and 24-hr reperfusion study [87]. However, whether cardiac fatty acid oxidation rates were actually decreased in this particular study was not determined.

### Glucose metabolism

As decreases in fatty acid oxidation result in a corresponding increase in glucose oxidation in the heart via the Randle cycle, several studies have explored the potential benefits to increasing cardiac glucose oxidation in ischemic heart disease. The pharmacological agent dichloroacetate (DCA) has proven to be a major tool for such studies, as it increases glucose oxidation rates via inhibiting PDHK isoforms. Direct treatment of isolated working rat or mouse hearts with DCA (1.5–3 mM) has been shown to improve the recovery of cardiac work (Joules *x* min^-1^ *x* g dry weight^-1^) following global no-flow ischemia [79,88], and this has also been observed in whole-body PDHK4 KO mice [88]. Likewise, treatment of mice with DCA (via intraperitoneal injection at 100 mg/kg and supplied in the drinking water at 110 mM) reduced infarct size following a 30-min temporary LAD coronary artery occlusion and 24-hr reperfusion protocol [88]. In contrast, PDH^CardiacKO^ mice developed larger infarct sizes following a 10-min temporary LAD coronary artery occlusion and 24-hr reperfusion protocol [89]. A limitation with the abovementioned studies is that these were all performed in nondiabetic animals, though DCA has been shown to increase glucose oxidation rates in isolated working hearts from diabetic rats [90], so it remains possible that stimulating glucose oxidation may be a viable approach to alleviating ischemic heart disease in T2D. In support of this, treatment of mice fed an HFD for 8–10 weeks with reconstituted high-density lipoprotein (HDL) increased *in vivo* cardiac glucose uptake assessed via ^18^FDG PET imaging 30-min post-LAD coronary artery occlusion, which was associated with a reduction in infarct size [91]. Furthermore, seahorse analysis also demonstrated increases in glucose uptake and glucose oxidation in isolated cardiomyocytes from these animals. Yet, it remains unknown whether the increase in cardiac glucose oxidation is responsible for the decreased infarct size, as there is potential for reconstituted HDL to interact with other targets (i.e., proteins) that could account for such observations. Future studies would need to assess whether treatment with reconstituted HDL can still limit infarct expansion in PDH^CardiacKO^ mice, since glucose oxidation cannot be stimulated in these animals.

### Ketone metabolism

Currently, there are limited preclinical studies interrogating whether manipulating cardiac ketone or amino acid metabolism can influence outcomes in ischemic heart disease, particularly in the context of T2D. Treatment of Langendorff perfused nondiabetic mouse hearts with 3-mM βOHB improved the recovery of LV developed pressure in response to a 30-min global no-flow ischemia and 40-min reperfusion protocol [92]. However, whether this benefit was attributed to increases in cardiac βOHB oxidation was not determined and, instead, may potentially stem from the ability of βOHB to inhibit nucleotide-binding domain-like receptor protein 3-mediated inflammation.

## Targeting cardiac substrate metabolism to treat DbCM

### Fatty acid metabolism

As studies in animals and humans strongly support that cardiac fatty acid oxidation is elevated in obesity/T2D, several studies have interrogated whether normalizing cardiac fatty acid oxidation can improve cardiac function in this setting. A 3-week treatment with the 3-KAT inhibitor, trimetazidine (15 mg/kg), alleviated the mild systolic and diastolic dysfunction observed in 26-week-old mice fed an HFD for 13 weeks [93]. This improvement in cardiac function was also associated with a reduction in cardiac hypertrophy, as evidenced by a decreased heart weight and LV mass normalized to tibial length. While reductions in cardiac fatty acid oxidation in whole-body MCD KO mice subjected to experimental obesity (via HFD feeding) were associated with improved insulin-stimulated cardiac glucose oxidation, no changes in *ex vivo* or *in vivo* cardiac function were observed [94]. However, there are well-documented challenges in using HFD models of obesity to cause consistent and reproducible cardiac dysfunction [32]. As such, it remains unknown whether pharmacological or genetic inhibition of MCD activity could benefit cardiac function in obesity/T2D if a more appropriate model was used. Of interest, pharmacological inhibition of aldose reductase with AT-001 decreased cardiac fatty acid oxidation rates, which was associated with an alleviation of diastolic dysfunction in mice subjected to the HFD/low-dose STZ model of T2D [95]. It remains unclear how inhibition of aldose reductase leads to a decrease in cardiac fatty acid oxidation, and whether these reductions in fatty acid oxidation are required for the beneficial actions on diastolic function. As previously stated, PPAR agonists (e.g., fibrates and thiazolidinediones) have been shown to decrease cardiac fatty acid oxidation via their systemic actions to decrease circulating lipid supply to the heart [84,85]. In support of this, treatment of Otsuka Long-Evans Tokushima Fatty rats for 5–10 weeks with the thiazolidinedione, pioglitazone, alleviated LV diastolic dysfunction, though cardiac fatty acid oxidation rates were not measured in this study [96,97].

Alleviating cardiac lipotoxicity has also been explored as a potential strategy to alleviate DbCM. For example, whole-body KO of ATGL results in robust cardiac lipid accumulation and a lipotoxic cardiomyopathy phenotype [98], while cardiac-specific ATGL overexpression has been shown to improve *in vivo* systolic and diastolic function in mice subjected to high-fat/high-sucrose diet-induced obesity [99]. Aging is also associated with increased body weight gain and risk for obesity/T2D, and aged whole-body CD36 KO mice exhibit robust improvements in *in vivo* systolic function compared with their WT littermates, which was associated with significantly decreased cardiac TAG content [100]. As previously stated, cardiac-specific PPARα transgenic mice exhibit a DbCM phenotype, and crossing these animals to whole-body CD36 KO mice alleviates their cardiac steatosis and restores LV ejection fraction (LVEF) when these animals are placed on an HFD for 4 weeks [101]. Furthermore, the introduction of a cardiac-specific LPL KO in cardiac-specific PPARα transgenic mice also alleviates their cardiac steatosis and restores LVEF when these animals are placed on an HFD for 4 weeks [102]. It was also demonstrated in the latter study that LPL-mediated lipolysis from very low-density lipoproteins (VLDLs) is required for the activation of PPARα transcriptional activity in neonatal rate ventricular cardiomyocytes, as these findings were nonexistent when LPL was present with TAG-depleted VLDL. Last, in support of a key role for GSK3α-mediated phosphorylation of PPARα at Ser280 as a mediator of lipotoxicity in DbCM, transgenic mice with a knock-in mutation of alanine at Ser280 (mimicking a PPARα that cannot be phosphorylated at Ser280) exhibited an amelioration of cardiac steatosis and diastolic dysfunction when fed an HFD for 8 weeks [51].

Of interest, the studies interrogating cardiac ATGL activity illustrate that increases in cardiac lipotoxicity may be more important toward diabetes-related cardiac dysfunction than perturbations in cardiac fatty acid oxidation rates. For example, both cardiac-specific ATGL overexpressing and cardiac-specific ATGL KO mice exhibit decreases in cardiac fatty acid oxidation, but cardiac dysfunction is only present in the latter, which is associated with increased cardiac TAG content and likely other lipotoxic mediators (e.g., ceramides) [99,103]. Nonetheless, a potential concern with using ATGL KO and cardiac-specific ATGL KO mouse models is that the cardiac lipid accumulation is massive and not really reflective of the extent that lipids accumulate in the myocardium in obesity/T2D. Hence, it is possible that the cardiac dysfunction in these models is secondary to an artificial lipid overload, whereby the excess lipid could directly interfere with the cardiomyocyte contractile and relaxation machinery. In support of this, many KO mouse models (e.g., MCD KO mice and PPAR KO mice) that result in decreased cardiac fatty acid oxidation combined with increased cardiac TAG/lipid accumulation paralleling that observed in obesity/T2D exhibit normal cardiac function [94,104]. Moreover, increased cardiac TAG content in humans with either obesity, prediabetes, or T2D is not associated with any overt systolic dysfunction [105].

### Glucose metabolism

Several preclinical studies have demonstrated that restoring cardiac glucose oxidation rates by enhancing PDH activity can ameliorate experimental DbCM. *In vivo* hyperpolarized [1-^13^C] pyruvate MRS revealed that treatment with DCA administered in the drinking water for 4 weeks restored cardiac PDH flux in male Wistar rats subjected to the HFD/low-dose STZ model of DbCM, which was associated with improved diastolic function (decreased E/e′ ratio) [60]. Because FoxO1 is a negative regulator of PDH activity via transcriptional regulation of *Pdk4*, it has been demonstrated that pharmacological FoxO1 inhibition via treatment with AS1842856 improved cardiac glucose oxidation and diastolic function (decreased E/e′ and increased e′/a′ ratios) in mice subjected to the HFD/low-dose STZ model of DbCM [106]. Supporting the notion that increases in cardiac PDH activity and glucose oxidation are required for FoxO1-mediated cardioprotection, AS1842856 treatment was unable to improve diastolic function in PDH^CardiacKO^ mice subjected to this model of experimental DbCM. Illustrating the translational potential of stimulating cardiac glucose oxidation as an approach for DbCM, glucagon-like peptide-1 (GLP-1) receptor (GLP-1R) agonists are a glucose-lowering drug class that improve cardiovascular outcomes in people with T2D [107], and these agents increase cardiac glucose oxidation rates in mice with HFD/low-dose STZ-mediated DbCM [58]. Similar to what was observed with pharmacological FoxO1 inhibition, treatment with the GLP-1R agonist, liraglutide, also failed to alleviate DbCM and diastolic dysfunction (decreased E/e′ and increased e′/a′ ratios) in PDH^CardiacKO^ mice with experimental T2D (HFD/low-dose STZ) [108]. A concern with interpreting the findings in PDH^CardiacKO^ mice may relate to the fundamental importance of PDH and glucose oxidation toward regulating cardiac function. It remains possible that if glucose oxidation is necessary to support normal diastolic function, no therapeutic strategy would impart benefit in PDH^CardiacKO^ mice. Arguing against this notion, unpublished findings from our laboratory have demonstrated that treatment with metformin, which acts independently of PDH and glucose oxidation, is able to alleviate diastolic dysfunction in PDH^CardiacKO^ mice with T2D.

Increases in mitochondrial calcium positively regulate PDH activity, and mitochondrial calcium uptake is diminished in T2D via increased expression of MCUb [67]. Interestingly, it has been demonstrated that gene therapy with a dominant-negative transgene of MCUb can restore cardiac mitochondrial calcium uptake, PDH activity, and glucose oxidation in mice subjected to HFD/low-dose STZ-mediated DbCM [67]. This was further associated with increased *in vivo* systolic function, while improving contractility and relaxation in isolated ventricular cardiomyocytes from these animals.

Because of the Randle cycle phenomenon, a potential concern with stimulating cardiac glucose oxidation to alleviate DbCM is that the corresponding decrease in fatty acid oxidation may promote cardiac lipid accumulation. Increases in cardiac lipid accumulation may exacerbate the lipotoxicity phenotype observed in DbCM, thereby impairing cardiac function and/or worsening cardiac insulin resistance. The majority of the abovementioned studies have not thoroughly assessed cardiac lipid species or cardiac insulin sensitivity in response to stimulating PDH activity and glucose oxidation, though FoxO1 inhibition in mice with DbCM did not increase cardiac TAG content [106]. Hence, further research in both animals and humans with T2D is needed to determine whether this is a real cause for concern with pursuing this metabolic strategy.

### Ketone and amino acid metabolism

Despite βOHB oxidation being decreased in several mouse models of T2D [74,75], pharmacological inhibition of SCOT activity and subsequent ketone oxidation improved several parameters of diastolic function (decreased E/e′ and left atrial size) in mice subjected to HFD/low-dose STZ-mediated DbCM. Moreover, treatment of *db*/*db* mice with a ketone ester (supplied in the drinking water) to elevate circulating ketone levels and presumably cardiac ketone oxidation improved both diastolic (increased mitral E/A ratio) and systolic function (increased fractional shortening). While these observations may seem at odds with each other, pharmacological inhibition of SCOT increases tissue accumulation of βOHB [109], and if ketone-mediated regulation of cell signaling is more relevant to cardioprotection in T2D/DbCM [23,24], it may explain why both approaches yielded beneficial outcomes.

Regarding amino acid metabolism, L-citrulline is a potent precursor for L-arginine synthesis and more readily absorbable than L-arginine, thus L-citrulline supplementation can increase nitric oxide levels by boosting L-arginine supply for nitric oxide synthase. Of interest, treatment of *db*/*db* mice with L-citrulline for 8 weeks improved both systolic (increased LVEF) and diastolic function (increased mitral E/A ratio), which was associated with increased circulating nitric oxide levels [110]. In other studies, glucosyringic acid has been demonstrated to suppress periostin activity, which increases BCAA catabolism in cardiac fibroblasts by increasing the expression of BCAA transaminase 2, thereby alleviating cardiac hypertrophy and cardiac dysfunction (increased LVEF) in mice subjected to an HFD/high-dose STZ (120 mg/kg) model of T2D [111].

## Targeting cardiac energy metabolism to treat heart failure in T2D

### Fatty acid metabolism

While T2D is associated with a distinct cardiac metabolic profile, there are some stark contrasts with the cardiac metabolic profile in heart failure. In particular, the failing heart is often referred to as an engine out of fuel, whereby ATP content can be decreased by upwards of 30% when compared with the healthy heart [112]. Thus, while cardiac fatty acid oxidation is elevated in T2D, many studies have observed that cardiac fatty acid oxidation is reduced in heart failure, though this is often dependent on disease severity [14]. These divergent metabolic phenotypes present a unique challenge with regard to cardiac fatty acid oxidation as a potential target, as people with T2D are at increased risk of or often comorbid for heart failure. Addressing these challenges, *db*/*db* mice exhibit improved outcomes (decreased LV mass, increased LVEF) in response to transverse aortic constriction (TAC)-induced heart failure [113]. The authors concluded that these salutary actions may be attributed to the fact that TAC leads to a decline in cardiac fatty acid oxidation [114], but this may have been abolished in *db*/*db* mice since the latter have well-documented increases in cardiac fatty acid oxidation [6].

### Glucose metabolism

While cardiac glucose uptake is often elevated in heart failure, cardiac glucose oxidation rates are generally decreased or remain unchanged in the setting of heart failure, which has been observed in animals with pacing-induced heart failure, TAC-induced heart failure, and angiotensin II infusion-induced heart failure [115–117]. In insulin-resistant Dahl salt-sensitive rats fed a high-salt diet, DCA treatment (80 mg/kg/day via the drinking water) for 7 weeks increased cardiac PDH activity, which was associated with improved survival, preserved systolic function (LV fractional shortening), decreased cardiac hypertrophy, and prevented the transition to a decompensated heart failure [118]. Likewise, in 13-month-old female mice fed an HFD containing Nω-nitro-L-arginine methyl ester (L-NAME) in the drinking water for 10 weeks, treatment with an experimental PDHK inhibitor, MMR-0053, increased LVEF and decreased cardiac hypertrophy, while improving survival [119]. As previously stated, GLP-1R agonists stimulate cardiac glucose metabolism [107], and GLP-1 infusion for 3 months also improves survival and LVEF, while decreasing cardiac hypertrophy in spontaneously hypertensive, heart failure-prone obese rats, though glucose oxidation rates were not assessed in this study [120].

### Ketone and amino acid metabolism

Several preclinical studies have alluded to the importance of increased circulating ketones and subsequent cardiac ketone metabolism as a potentially adaptive response in heart failure [23], though this is often not considered in the context of T2D. However, administration of an oral ketone ester (1 mg/g) for 30 days in mice fed an HFD for 13 months and treated with desoxycorticosterone pivalate during the final month to induce heart failure with preserved ejection fraction produced robust increases in circulating βOHB levels [121]. Intriguingly, this led to decreased lung edema and circulating brain natriuretic peptide levels, while improving exercise performance and diastolic relaxation, though it was not determined whether these actions were dependent on the metabolic versus signaling actions of ketones. Despite the above-described model being developed to induce heart failure with preserved ejection fraction, the HFD component induces insulin resistance and hyperglycemia, suggesting that such a metabolic approach may also have utility in heart failure with coexistent T2D.

Metabolomics studies have demonstrated key links between perturbations in BCAA metabolism and coronary artery disease and/or heart failure [122,123]. Preclinical studies support these links and have shown that defects in cardiac BCAA oxidation contribute to heart failure [124], though whether this is translatable to heart failure with coexistent T2D remains to be determined.

## Clinical studies targeting cardiac substrate metabolism in T2D

Despite preclinical studies demonstrating promise in the realm of targeting cardiac substrate metabolism as an approach to alleviate cardiovascular diseases (i.e., ischemic heart disease, DbCM, and heart failure) associated with T2D, this has been minimally explored in humans. Trimetazidine is the primary metabolic agent decreasing fatty acid oxidation rates that is used clinically in the management of cardiovascular disease. While it has been reported that the benefits of trimetazidine in heart failure may have salutary actions on cardiac function in overweight individuals [125], or in individuals with ischemic cardiomyopathy and T2D [126], overall patient sample sizes have been small. It also needs to be considered that the reduction in fatty acid oxidation with trimetazidine treatment in humans is quite mild, with PET imaging studies demonstrating a ~10% reduction [125]. With the majority of preclinical and clinical studies observing robust increases in cardiac fatty acid oxidation in T2D, whether a 10% reduction in fatty acid oxidation rates would meaningfully improve diastolic and/or systolic function remains unknown. The experimental aldose reductase inhibitor, AT-001, also decreases cardiac fatty acid oxidation in preclinical models of DbCM [95], though studies in humans with DbCM and impaired exercise capacity have shown that treatment with AT-001 for 15 months did not improve peak O_2_ consumption or exercise tolerance [127]. Nevertheless, it remains to be determined whether AT-001 can decrease cardiac fatty acid oxidation in humans with T2D as it does in animals.

With regard to glucose metabolism, DCA has been the most extensively studied pharmacological agent that can stimulate cardiac glucose oxidation, though studies in humans are limited due to DCA’s very short half-life requiring constant infusion to achieve optimal PDHK inhibition [128]. Studies have shown that short-term IV infusion of DCA can improve hemodynamic parameters in individuals with congestive heart failure [129], but once again patient sizes were small and whether this is translatable to those with heart failure and T2D remains unknown. As GLP-1R agonists have been shown in several large-scale trials to improve cardiovascular outcomes in people with T2D [107], while also stimulating cardiac glucose oxidation in preclinical studies [58,130], they may represent a more feasible approach to modifying cardiac glucose metabolism. However, ^18^FDG PET imaging studies in humans with heart failure with reduced ejection fraction did not observe increases in cardiac glucose utilization in response to treatment with albiglutide [131]. Since PET imaging with ^18^FDG is only a measure of glucose uptake and not glucose oxidation, hyperpolarized ^13^C MRS studies would be a better approach to determine whether GLP-1R agonists increase cardiac glucose oxidation in humans with T2D as they do in animals. As GLP-1R agonists are also frequently associated with decreases in circulating lipids (i.e., free fatty acids and TAGs), it would be important to determine via PET imaging whether these agents decrease cardiac fatty acid oxidation rates.

The sodium-glucose cotransporter-2 (SGLT2) inhibitors are another drug class for T2D that have also been shown to improve cardiovascular outcomes [132], and a popular hypothesis to explain their cardioprotective actions stems from their ability to increase circulating ketones [23,133]. Since increases in cardiac ketone oxidation in heart failure are thought to be an adaptive response, it has been reasoned that increasing ketone delivery to the heart in T2D with SGLT2 inhibitors would increase ketone oxidation and alleviate cardiac dysfunction. Unfortunately, there have not been conclusive studies performed in humans to date demonstrating that treatment with SGLT2 inhibitors in T2D leads to an increase in cardiac ketone oxidation rates, due, in part, to lack of characterized tracers that can be used to assess flux through ketone oxidation with either PET imaging or ^13^C MRS. In addition, studies using isolated working hearts from *db*/*db* mice observed that treatment with empagliflozin did not increase ketone oxidation rates. A limitation with these studies that needs to be considered is that ketone concentrations in the perfusate were fixed in all experimental groups. As such, this does not rule out that these agents may increase cardiac ketone oxidation in humans with T2D, since circulating ketone concentrations are a key determinant of ketone oxidation and is increased in response to treatment with SGTL2 inhibitors [23]. More recently, the EMPA-VISION (Assessment of Cardiac Energy Metabolism, Function and Physiology in Patients with Heart Failure Taking Empagliflozin) study also reported that treatment with empagliflozin did not affect cardiac energetics, as determined by no change in the cardiac phosphocreatine/ATP ratio using binuclear (^31^P and ^1^H) MRS [134]. Decreased enthusiasm with this metabolic hypothesis has led to a shift in views regarding how SGLT2 inhibitors may elicit cardioprotection in T2D, with recent attention focused on the induction of autophagy [135].

## Conclusions, caveats, and future considerations

A plethora of evidence in both animal and human studies support that both obesity and/or T2D result in robust changes to cardiac substrate metabolism. These perturbations in substrate metabolism are not simply passengers in the progression of disease, but it is likely that they directly contribute in some shape or form to cardiac pathology in T2D. As such, numerous preclinical studies have illustrated that correcting these perturbations can improve cardiac function in models of T2D.

Nonetheless, there are important caveats that need to be taken into consideration with this evidence. This includes the major limitation that the vast majority of animal studies in this area have predominantly been in males, and as there are sex-specific differences in cardiac metabolism [136,137], it will be imperative for future cardiac metabolism studies in animals with T2D to incorporate female animals. Another relevant concern is that many in the field often use the HFD-induced model of obesity to study cardiac dysfunction in the context of insulin resistance/prediabetes. While this is undoubtedly a valid model to study the pathological mechanisms responsible for obesity, insulin resistance, prediabetes, and/or T2D [31], this model is highly inconsistent in its ability to produce cardiac dysfunction [32]. Indeed, studies in the early 2000s often reported marked declines in LVEF with HFD-induced obesity, reflective of a heart failure with reduced ejection fraction phenotype, but more frequent reports are appearing that observe negligible cardiac dysfunction (both systolic and diastolic) with HFD-induced obesity. It is difficult to reconcile these discrepancies, but it may stem from the fact that ultrasound and cardiac MRS imaging technologies for rodents have significantly improved since these original studies were first reported. Furthermore, many of these studies utilized ultrasound echocardiography systems designed for use in humans (e.g., ACUSON Sequoia systems), versus those specifically designed for small rodent use (e.g., VisualSonics systems) that the majority of researchers in the field are now using.

Moving forward, it will be important for future studies to incorporate models of ischemic heart disease (e.g., LAD coronary artery occlusion) and heart failure (e.g., TAC) in animals subjected to models of obesity and/or T2D. Aside from the study incorporating TAC in *db*/*db* mice [113], there is a lack of thorough studies assessing cardiac substrate metabolism in T2D in the context of ischemia/reperfusion or heart failure. It is understandable from a feasibility standpoint that the complexity this adds toward overall research design has led to a lack of these types of studies. However, they are required in order to determine whether targeting cardiac substrate metabolism has therapeutic potential, since many people living with T2D die from ischemic heart disease or heart failure. Another potential factor future studies will need to consider relates to the aspect of thermoneutrality, which, for a mouse, lies in the range of 27–30°C, though most mouse studies are performed at room temperature. Increasing evidence illustrates that energy metabolism differs in mice at room temperature versus thermoneutrality, and how cardiometabolic disorders progress in mice in response to HFDs [138].

Taking these factors into consideration with future studies of cardiac substrate metabolism in T2D should help further advance our knowledge of how perturbations in cardiac substrate metabolism contribute to T2D-related cardiovascular diseases. Despite the potential promise in targeting cardiac substrate metabolism to alleviate cardiovascular disease in T2D, whether this benefit will translate to humans is an open question requiring further research that hopefully will be answered in the years to come. Because there are clear differences in how T2D versus heart failure affect cardiac substrate metabolism, there will also be key challenges that need to be considered with pursuing metabolic strategies to improve cardiovascular health in people with T2D. It remains possible that specific metabolic approaches (e.g., decreasing cardiac fatty acid oxidation) may yield salutary actions in people with T2D in the absence of heart failure but actually worsen outcomes in people with T2D and comorbid heart failure. These differences will need to be factored into clinical trial design as new metabolic therapies are developed.

## Abbreviations

ACAT: acetoacetyl CoA thiolase
ATGL: Adipose tissue TAG lipase
ATP: adenosine triphosphate
BCAA: Branched-chain amino acid
BDH1: βOHB dehydrogenase
CPT1: carnitine palmitoyltransferase 1
CoA: coenzyme A
DCA: Dichloroacetate
DbCM: coenzyme A (CoA), diabetic cardiomyopathy
FACS: Fatty acyl CoA synthetase
FADH2: flavin adenine dinucleotide
18FDG: 18F-2-fluoro-2-deoxy-D-glucose
FoxO1: Forkhead box O1
GSK3α: Glycogen synthase kinase 3α
HDL: high-density lipoprotein
HFD: High-fat diet
3-KAT: 3-ketoacyl CoA thiolase
KO: knockout
LAD: left anterior descending
L-NAME: Nω-nitro-L-arginine methyl ester
LPL: lipoprotein lipase
LV: left ventricular
LVEF: Left ventricular ejection fraction
MCD: malonyl CoA decarboxylase
MCT: monocarboxylic acid transporter
MCUb: Mitochondrial calcium uniporter complex inhibitory subunit
MPC: mitochondrial pyruvate carrier
MRS: magnetic resonance spectroscopy
NADH: nicotinamide adenine dinucleotide
PDH: pyruvate dehydrogenase
PDHK: PDH kinase
PDHP: PDH phosphatase
PET: Positron emission tomography
PPARα: Peroxisome proliferator-activated receptor-α
ROS: reactive oxygen species
SCOT: Succinyl CoA:3-ketoacid CoA transferase
STZ: streptozotocin
TAC: Transverse aortic constriction
TAG: triacylglycerol
TCA: tricarboxylic acid
T2D: type 2 diabetes
VLDL: very-low density lipoproteins
βOHB: β-hydroxybutyrate
